# Supplementary material for: Implementation of basic package of care improved socio-economic conditions of lymphedema patients in rural Mali after two years follow-up
Source: PLOS Glob Public Health. 2025 Nov 11;5(11):e0005454. doi: 10.1371/journal.pgph.0005454 (PMC12604757; doi:10.1371/journal.pgph.0005454)
Supplement: S1 Checklist — (DOCX) [file pgph.0005454.s001.docx]

Inclusivity in global research

PLOS’ policy on inclusivity in global research aims to improve transparency in the reporting of research performed outside of researchers’ own country or community and ensures that PLOS publications reporting global research adhere to high standards for research ethics and authorship. Authors of relevant research articles may be asked to complete the questionnaire below, which outlines ethical, cultural, and scientific considerations specific to inclusivity in global research. This questionnaire may be requested when researchers have travelled to a different country to conduct research, if research uses samples collected in another country, research with Indigenous populations or their lands, or if research is on cultural artefacts. Researchers travelling to another country solely to use laboratory equipment will not normally be required to complete the questionnaire. However, the questionnaire can be requested at the journal’s discretion for any submission – if you have been requested to complete this questionnaire by the PLOS journal you submitted to, please do so.

Please complete the questionnaire below and include this as a Supporting Information file with your manuscript. Note that if your paper is accepted for publication, this checklist will be published with your article in the supporting information files. Please ensure that you reference the checklist in the main body of your manuscript. We suggest adding a subsection ‘Inclusivity in global research’ to your Methods section and adding the following sentence: “Additional information regarding the ethical, cultural, and scientific considerations specific to inclusivity in global research is included in the Supporting Information (SX Checklist)”

The questions have been designed to be applicable to a wide range of study types, and there are subsections for both human subjects research and non-human subjects research. If any of the questions are not relevant to your research please mark them as “N/A” as appropriate.

**Ethical considerations, permits and authorship**

*This section is applicable to all research types.*

Provide details as to who granted permissions and/or consent for the study to take place in the Methods section of your manuscript. This should include the names of **all** ethics boards, governmental organizations, community leaders or other bodies that provided approval for the study. If individuals provided approval refer to these people by their role or title but do not list their name(s).

Reported on page number: 5-6

This study was approved by the Ethics Committee of the University of Science, Techniques and Technology of Bamako (USTTB), Mali (Approval Number: 2021/231/USTTB and signed by the president of IRB). In Mali we generally get approval from the community leader orally during the pre study meeting with the community leaders.

We acknowledge the collaboration of the National Lymphatic Filariasis Elimination Program and the health districts of Kolondieba and Kolokani. During the implementation of study work with the health authorities in charge of the different study sites.

The materials and communication were provided in the local language. The manuscript specifies that "Participants received full verbal explanations of the study objectives, procedures, risks/benefits, and their right to withdraw without penalty, in the local language (Bambara)". Additionally, the standardized survey questionnaire was "pre-tested, corrected, and validated by the research team before being used in the field by trained interviewers". Interview guides for in-depth interviews (IDIs) and focus group discussions (FGDs) were also "developed, pre-tested, edited and validated during a workshop in Bamako before being administered in the field"

If there were any deviations from the study protocol after approval was obtained please provide details of these changes in the Methods section of your manuscript.

Reported on page number: 6

any deviations were observed during this cross sectional study and the protocol was implemented in adherence with the approved protocol by the USTTB IRB. The ethic committee approval letter was shared as an appendix to the manuscript.

Did this study involve local collaborators that are residents of the country where the research was conducted or members of the community studied? If you do not have any authors from said communities, please provide an explanation for this below.

Most of the authors listed are local Malian researchers except Thomas B. Nutman.

Everyone listed as an author should meet PLOS’ criteria for authorship and all individuals who meet these criteria should be included in the author byline, rather than the acknowledgements. For further information please see the journal’s Authorship Policy.

**Human subjects research (e.g. health research, medical research, cross-cultural psychology)**

Did you obtain written informed consent from a representative of the local community or region before the research took place? How did you establish who speaks for the community? Details of written informed consent obtained from study participants should be reported separately in the Methods section of your manuscript.

The study protocol has been approved by the Ethics Committee of the University of Science, Techniques and Technology of Bamako (USTTB), Mali. The survey was conducted in different rural villages of Mali. To minimize risks for the interviewers, they were supported for community entry by community helath workers and local guide. Orall consent was obtained from a representative of each local community. The study did obtain written informed consent from all individual participants. For participants unable to write, fingerprint consent was obtained in the presence of a witness.

How did members of the local community provide input on the aims of the research investigation, its methodology, and its anticipated outcome(s)?

Data collection was conducted in face-to-face interviews mode using a questionnaire designed according to the research objectives. The questionnaire was developed with the Kobocollect application and installed on mobile devices.

When engaging with the local community, how did you ensure that the informed consent documents and other materials could be understood by local stakeholders?

A report was share with the USTTB the funder of Study. Additionally, the research findings will be shared at conferences held in Mali, attended by key decision-makers. In this context the data was presented during American Society of Tropical Medicine and Hygiene2024 Annual Meeting in November 13-17, 2024

In New Orleans. After the survey, the study team conducted field visit to share the finding with the differences communities of the study.

Will the findings of the research be made available in an understandable format to stakeholders in the community where the study was conducted (e.g. via a presentation, summary report, copies of publications, etc.)? Please provide details of how this will be achieved.

**Non-human subjects research using specimens/ animals collected as part of the study, or those housed in archival collections. Examples include archaeology, paleontology, botany and zoology.**

Did the permission you obtained from a local authority to perform the study include an agreement on access to outputs and benefit sharing? This may include procedures to enable fair distribution of the benefits and resources arising from the research performed. Please include any details of Prior Informed Consent and Benefit Sharing Agreements obtained. These may be required by field-specific regulations, for example the Convention on Biological Diversity (CBD) and the associated Nagoya Protocol.

N/A

If the material used in your study was imported, please A) provide the year it was imported and B) indicate whether permits were obtained to import/export the materials used, C) provide details of any permits obtained. If this information is not available, please indicate this.

N/A

If you used archival specimens, please state how the material used in your study was acquired by the institute it is held in and provide details of any permits obtained for the original excavations/ sample collection. If this information is not available, please indicate this.

N/A

How was the potential cultural significance of the materials collected in your study to local communities considered in your research design? Were Indigenous peoples and/or local researchers and institutions involved with archaeological excavations / collection of specimens? If so, please provide a description of their involvement.

N/A

If your manuscript includes photographs of human remains please indicate whether authors obtained permission from descendants or affiliated cultural communities to do so.

N/A
