# Supplementary material for: Implementation of basic package of care improved socio-economic conditions of lymphedema patients in rural Mali after two years follow-up
Source: PLOS Glob Public Health. 2025 Nov 11;5(11):e0005454. doi: 10.1371/journal.pgph.0005454 (PMC12604757; doi:10.1371/journal.pgph.0005454)
Supplement: Attachment — Submitted filename: PGPH-D-25-00707.pdf [file pgph.0005454.s002.pdf]

PLOS Global Public Health

Implementation of Basic Package of Care Improved Socio-Economic Conditions of Lymphedema Patients in rural Mali after two years follow-up.

--Manuscript Draft--

**Manuscript Number:**	PGPH-D-25-00707
**Article Type:**	Research Article
**Full Title:**	Implementation of Basic Package of Care Improved Socio-Economic Conditions of Lymphedema Patients in rural Mali after two years follow-up.
**Short Title:**	Improved Care lead to Improved Lives for Lymphedema Patients in Rural Mali
**Corresponding Author:**	Housseini Dolo, M.D.<br>Neglected Tropical Diseases Research and Training Unit, International Center of Excellence in Research, University of Sciences, Techniques and technologies of Bamako, Mali<br>Bamako, MALI
**Order of Authors:**	Housseini Dolo, M.D., PhD
	Diadje Tanapo, MD, MPH
	Sekou Oumarou Thera, MD, MPH
	Moussa Sangare, MD, MPH
	Abdoul Fatao Diabaté, MD, MPH
	Lamine Diarra, MD, MPH
	Salif Seriba Doumbia, MD, MPH
	Siaka Yamoussa Coulibaly, MD, MPH
	Ilo Dicko, MD, MPH, PhD
	Oumar Coulibaly, MD
	Ousmane Faye, MD, PhD
	Hamadoun Sangho, MD, DrPH
	Seydou Doumbia, MD, PhD
	Yaya Ibrahim Coulibaly, MD, MPH, PhD
	Thomas B. Nutman, MD
**Keywords:**	Impact, hygiene, socio-economic, lymphedema, Kolondieba, Kolokani and Mali
**Abstract:**	A local hygiene package has previously been demonstrated to be effective at improving lymphedema (LE) patient's quality of life. This study aimed to assess the socio-economic impact of regular local care based on the hygiene package used among LE patients.<br>We conducted a cross-sectional study using a before-and-after approach to assess the effect of basic package of care for LE management among LE patients in the health districts of Kolondieba and Kolokani from August to December 2021. Quantitative data were collected using a digital questionnaire in Kobotoolbox. These data were analysed using SPSS version 25.0. We used a pre-established interview guide to conduct in-depth interviews and focus group discussions. We performed a thematic analysis approach using Quirkos version 2.<br>We investigated 196 LE patients with a median age of 56 years [25-70]. We observed a reduction in the monthly frequency of acute filarial attacks (ADLA) from 90.8% (178/196) before the trial to 43.9%, (86/196) after the trial (p<10-3). Importantly, the average ADLA attack management cost decreased significantly from US$20.01 before the trial to US$5.7, after the trial (p<10-3). Patients reported that the hygiene program reduced social isolation and stigma and improved their ability to work.<br>This study suggests that the use of a basic package of care significantly improved the

Powered by Editorial Manager® and ProduXion Manager® from Aries Systems Corporation

	socioeconomic conditions of LE patients mainly by reducing the cost of ADLA attacks management. Therefore, a community-based lymphedema management program is needed with income-generating activities its sustainability.
**Additional Information:**	
**Question**	**Response**
**Financial Disclosure**<br><br>Enter a financial disclosure statement that describes the sources of funding for the work included in this submission and the role the funder(s) played. This includes grants and any commercial funding of the work or authors.<br><br>This statement will be typeset if the manuscript is accepted for publication.<br><br>*Please review the submission guidelines and the instructions link below for detailed requirements and guidance.*	This study was funded by the University of Sciences, Techniques and Technologies of Bamako through the research promotion grant to the teaching and research professors awarded to HD. In addition, the study was funded in part (to TBN) by the Division of Intramural Research (DIR) of the National Institute of Allergy and Infectious Diseases, NIH.
**Competing Interests**<br><br>On behalf of all authors, disclose any competing interests that could be perceived to bias this work.<br><br>This statement will be typeset if the manuscript is accepted for publication.<br><br>*Please review the instructions link below and PLOS Global Public Health's competing interests policy to determine what information must be disclosed at submission.*	The authors declare that they have no conflict of interests.
**Data Availability**<br><br>Before publication, Authors are required to make fully available and without restriction all data underlying their findings. Please see our PLOS Data Policy page for detailed information on this policy.	The data used in this manuscript can be found through the International Center of Excellence in Research of the University of Science, Techniques and Technologies of Bamako by sending an email to the principal investigator and first author of the manuscript.

A **Data Availability Statement**, detailing where the data can be accessed, is required at first submission. Insert your Data Availability Statement in the box below.<br><br>Please see the data reporting section of our submission guidelines for instructions on what you need to include in your Data Availability Statement.<br><br>This statement will be typeset if the manuscript is accepted for publication.<br><br>*PLOS allows rare exemptions to address legal and ethical concerns. If you have legal or ethical restrictions, please use the box below to detail these in full sentences for the Journal team to consider.*	

Powered by Editorial Manager® and ProduXion Manager® from Aries Systems Corporation

Manuscript

Click here to access/download;Manuscript;h Dolo et al LE socio Economic main document_final-revised.docx

Title

Implementation of Basic Package of Care Improved Socio-Economic Conditions of Lymphedema Patients in rural Mali after two years follow-up.

Short title

Improved Care lead improved Lives for Lymphedema in Mali's Rural Communities

Authors

Housseini Dolo[1,3]*, Diadje Tanapo[1,&], Sekou Oumarou Thera[1,&] , Moussa Sangaré[1,3] , Abdoul Fatao Diabaté[1] , Lamine Diarra[1] , Salif Sériba Doumbia[1] , Siaka Yamoussa Coulibaly[1], Ilo Dicko[1] , Oumar Coulibaly[1] , Ousmane Faye[2] , Hamadoun Sangho[3] , Seydou Doumbia[3], Yaya Ibrahim Coulibaly[1,2] , Thomas B. Nutman[4]

Affiliations

[1] Neglected Tropical Diseases Research and Training Unit, International Center of Excellence in Research, University of Sciences, Techniques and Technologies of Bamako, Bamako, Mali.

[2] Dermatology Hospital of Bamako, Bamako, Mali

[3] Department of Teaching and Research in Public Health and Specialties, Faculty of Medicine and Odonto Stomatology of Bamako, University of Sciences, Techniques and Technologies of Bamako, Bamako, Mali.

[4] Laboratory of Parasitic Diseases, National Institute of Allergy and Infectious Diseases, National Institutes of Health, Bethesda, MD, USA.

[&]Contributed equally as second co-authors

*Corresponding author: Dr Housseini Dolo, MD, PhD, email: hdolo@icermali.org

Abstract

A local hygiene package has previously been demonstrated to be effective at improving lymphedema (LE) patient's quality of life. This study aimed to assess the socio-economic impact of regular local care based on the hygiene package used among LE patients.

We conducted a cross-sectional study using a before-and-after approach to assess the effect of basic package of care for LE management among LE patients in the health districts of Kolondieba and Kolokani from August to December 2021. Quantitative data were collected using a digital questionnaire in Kobotoolbox. These data were analysed using SPSS version 25.0. We used a pre-established interview guide to conduct in-depth interviews and focus group discussions. We performed a thematic analysis approach using Quirkos version 2. We investigated 196 LE patients with a median age of 56 years [25-70]. We observed a reduction in the monthly frequency of acute filarial attacks (ADLA) from 90.8% (178/196) before the trial to 43.9%, (86/196) after the trial (p<10-3). Importantly, the average ADLA attack management cost decreased significantly from US$20.01 before the trial to US$5.7, after the trial (p<10-3). Patients reported that the hygiene program reduced social isolation and stigma and improved their ability to work.

This study suggests that the use of a basic package of care significantly improved the socioeconomic conditions of LE patients mainly by reducing the cost of ADLA attacks management. Therefore, a community-based lymphedema management program is needed with income-generating activities its sustainability.

Keywords: Impact, hygiene, socio-economic, lymphedema, Kolondieba, Kolokani and Mali

Introduction

People living with lymphedema (LE) experience attacks of acute adenolymphangititis (ADLA) that cause severe pain and loss of productivity (1,2). Consequently, these individuals are often unable to perform daily activities such as fetching water, harvesting crops, and performing certain household tasks. This contributes to reduced productivity within their community (3,4).

In 2021, the World Health Organization (WHO) estimated that approximately 40 million people suffer from the stigma and disabling clinical manifestations of lymphatic filariasis (LF), of which 15 million have LE (5). In 2016, a study reported by Dolo et al. identified 557 cases of LE in three previously LF-endemic districts in Bougouni, Kolokani, and Kolondieba (6).

Local hygiene basic package of care is important to reduce oedema progression and prevent associated superinfection that causes ADLA(7,8). However, ADLA management requires complex care that generates high costs resulting in loss of income for affected individuals and their families who usually are already facing poverty (9,10).

In the LF elimination strategy, WHO advocates burden reduction through the implementation of a basic package of care for LE management. This is one of the main components of the morbidity management and disability prevention program (MMDP), a second pillar of LF elimination as a public health problem (11). In this context, the Global LF Elimination Program (GPELF) was launched in 2000 to eliminate the disease as a public health problem by 2030 to coordinate prevention and case management activities (11). In Mali, based on a previous study on LE in three health districts (12), the majority of people with LE were living in remote rural areas.

In 2018, a multicentre study (LEDoxy NCT02927496 registered on 7 Oct 2016) was conducted in Mali, India, and Sri Lanka on the efficacy of doxycycline (compared to placebo) in addition to local hygiene in the early stages of LE (13). During LEDoxy, all subjects received hygiene kits and training on self-management of LE as recommended by WHO (14). Patients were also allowed to consult the nearest health centre gratis when there were any medical needs between two visits of the research team. Thirty-six months after the study completion, the current study aimed to assess the socio-economic impact of implementing a basic package of care in the management of LE in the two rural study health settings to fill the knowledge gap on the socio-economic impact of the WHO-recommended basic package of care in remote rural communities.

Methods

Ethical considerations

The protocol was approved by the Ethics Committee of the University of Sciences, Techniques, and Technologies of Bamako (USTTB) under the number 2021/231/USTTB. Participants' concerns about confidentiality were addressed by limiting access to data to the study investigators. Participants were informed of the purpose of the study, how their data would be used, and of their right to refuse to participate or to leave the study at any time. Informed consent was obtained from each participant before data collection.

Study sites and design

A cross-sectional study using a before-and-after approach was conducted for data collection from 26 September 2021 to 25 December 2021, using mixed methods (quantitative and qualitative) data collection. The study took place in the health districts of Kolondieba and Kolokani in Mali to assess post-lymphedema management effects on socio-economic indicators.

Study population

The surveyed persons were LE carriers enrolled in the doxycycline trial from June 27, 2018, to December 19, 2020. These health districts were chosen due to their endemicity for LF (12) and many recorded cases of LE that were closely followed as part of the LEDoxy clinical trial (15).

All participants agreed to voluntarily participate in the survey. We included the former LEDoxy study participant and were living in the same areas over the previous six months at least. For the quantitative component of this study, we used exhaustive sampling by inviting all the 220 patients from the LEDoxy study in the socioeconomic study. For the qualitative component of the study, convenience sampling was used. Thus, four focus group discussions (FGDs) and five individual in-depth interviews (IDIs) per health district were conducted with previous LEDoxy patients who volunteered. A total of eight FGDs and ten IDIs were planned.

Questionnaire design and administration

A survey questionnaire was ~~conceived~~ and was entered into the Kobo toolbox platform to collect quantitative data using smartphones. Questions related to patients' socio-economic conditions, sustainability of limb hygiene, and aids obtained for this disease management were included. This questionnaire was pre-tested, corrected, and validated by the research team before being used in the field by trained interviewers. Field collected data were checked and edited in real-time by the supervisors and the data manager using the Kobotoolbox platform that is available remotely using internet.

For IDIs and FGDs, interview guides were developed, pre-tested, edited and validated during a workshop in Bamako before being administered in the field. ~~These guides helped interviewers to collect qualitative data~~. Collected data were reviewed daily by the principal investigator to correct for accuracy and completeness or to address any identified issues to ensure the quality of the collected data.

For the economic evaluation of the data related to the management of one ADLA attack per month, we considered the costs reported by patients. First, the cost of managing one ADLA attack per month was estimated for each patient. Then, the amounts reported by patients were collected to form a dataset. To ensure the reliability of the analysis, extreme values were excluded (a total of 18 data). This exclusion of extreme values enabled us to obtain a more representative and reliable estimate of the average cost of managing one ADLA attack per month.

Data analyses

The Statistical Package for Social Sciences (SPSS 25.0) software was used to clean and analyse quantitative data. Categorical variables were summarized as proportion while continuous variables were presented as mean ± standard deviation. Comparisons of proportions were made using Pearson's Chi^2 or Fisher's exact test depending on their applicability. For comparison of continuous variables, Wilcoxon signed Rank test was used. Any p values =<0.05 were considered statistically significant. We compared the cost of managing ADLA episode before and 16 months after the local hygiene package implementation as part of LEDoxy. For qualitative data, records were captured and coded as themes and subthemes, and Quirkos software version 2 was used to support data analysis.

Results

Sociodemographic characteristics of LE patients after LEDoxy

A total of 196 patients were included in this study. The health district of Kolondieba had the largest number of participants with 72.4% (142/196). Females represented 86.7% (170/196) of the participants. The median age of the participants was 56 years [25-70]. More than half of the patients were married (59.7% (117/196)); most of them were not educated 90.8% (178/196), and 79.1% (155/196) were farmers (Table 1).

Table 1. Description of patients affected by LE 16 months after LEDoxy in the health districts of Kolondieba and Kolokani in 2021

Socio-demographic characteristics	Health districts		Total
	**Kolokani**	**Kolondieba**	
	n =54	n =142	**N=196**
	n (%)	n (%)	n (%)
**Gender**			
Female	43 (79.6)	127 (89.4)	170 (86.7)
Male	11 (20.4)	15 (10.6)	26 (13.3)
**Age group**			
Under 56 years old	21 (38.9)	76 (53.5)	97 (49.5)
56 years old and above	33 (61.1)	66 (46.5)	99 (50.5)
Median age [Min-Max]	60 years [30-70]	54 years [25-70]	56 years [25-70]
**Level of education**			
Schooled	4 (7.4)	14 (9.9)	18 (9.2)
Unschooled	50 (92.6)	128 (90.1)	178 (90.8)
**Marital status**			
Single	1 (1.8)	4 (2.8)	5 (2.5)
Married	34 (63)	83 (58.5)	117 (59.7)
Widow(er)	19 (35.2)	55 (38.7)	74 (37.8)
**Profession**			
Shopkeeper	1 (1.9)	4 (2.8)	5 (2.6)
Cultivator	39 (72.2)	116 (81.7)	155 (79.1)
Teacher	0 (0)	1 (0.7)	1 (0.5)

Housekeeper	11 (20.4)	13 (9.2)	24 (12.2)
Other	3 (5.5)	8 (5.6)	11 (5.6)

Min-Max: Minium-Maximum

LE-related difficulties and regularity of limb washing 16 months after LEDoxy

The proportions of patients who reported not having received help from a relative or friend in the village for the management of ADLA crises in Kolokani and Kolondieba health districts were 94.4% (51/54) and 88% (125/142) respectively. The proportions of patients who did not experience LE-related stigma or discrimination after LEDoxy study in Kolokani and Kolondieba health districts were 94.4% (51/54) and 82.4% (117/142) respectively. The proportions of patients reporting continued limb washing after LEDoxy in the two districts were 78.2% (111/142) in Kolondieba and 83% (45/54) in Kolokani (Table 2).

Table 2. Frequencies of social, support, and hygiene practices related constraints within LE-affected patients in the two health districts in 2021

	**Health districts**		
	**Kolokani**	**Kolondieba**	**p**
	**n (%)**	**n (%)**	
**Received support / assistance for LE**			
No	51 (94.4)	125 (88)	0.29
Yes	3 (5.6)	17 (12)	
**Social difficulties related to LE**			
No	51 (94.4)	117 (82.4)	0.03
Yes	3 (5.6)	25 (17.6)	

**Continuity of limb washing practices**			
No	9 (16.7)	31 (21.8)	0.55
Yes	45 (83.3)	111 (78.2)	

Changes in ADLA management cost

The average cost of managing an ADLA attack decreased significantly from 13,481 FCFA (US $20.01) before the introduction of basic package of care to 3,843 FCFA (US $5.7) after its implementation for 2 years, making an average decrease of 9,638 FCFA (US $14.3) (p<10-3) (Figure 1).

[Insert figure 1 here]

Fig 1 [Reduction on the average expenses for ADLA crisis management within lymphedema patients before and after the use of hygiene measures package in the health districts of Kolondieba and Kolokani in 2021]

Lymphedema patients' productivity

LE can have a significant impact on the productivity of affected individuals. Swelling of the arms or legs can make it difficult to perform daily duties. Treatment of LE often involves the use of compression bandages, which can help reduce swelling and improve mobility. However, this condition can still result in missed workdays and reduced productivity. This can have a negative impact on the socio-economic status of those affected and their families. The main themes that were discussed were the inability to work, particularly during episodes of ADLA.

A woman explained that she was not able to do any expected farming or other field work and that this necessarily resulted in a loss of income.

"*Our main source of income is farming and gardening; this disease prevents us from carrying out these different activities. So, we have almost no other source of income*." [FGD with elderly women in Kebila, Kolondieba]

Another participant said that before her disability, she used to farm and do small-scale horticulture, but with the acute crises, this is no longer possible.

"*I used to grow peanuts to earn money, but now I can't do it because of the acute crises*". [IDI with elderly women in Kebila, Kolondieba].

Patients with LE mentioned working hard, walking long distances, and being in contact with water, especially in winter, as factors that induced ADLA. This is illustrated by the following quote:

"*During the study, things were much better because we were able to work during the rainy season, but since the study was stopped, it has become difficult, if not impossible, again*" [FGD with elderly men from Kebila, Kolondieba].

Some participants reported that they were less productive than peers of their age group in the village. For example, a woman during an interview stated that:

"*With this disease, I can no longer produce shea butter or néré powder of "Parkia biglobosa" while all my age mates in the village do it to earn money*." [IDI with a woman from Kolondieba].

A group of LE carriers stated that the economic impact does not affect only the patient but also their close relatives.

"*I don't work anymore and sometimes I find it hard to ask the children for money. Sometimes, I used to take loans from people in order to pay for my medicine during crises, but, since LEDoxy, I can go anywhere and work."* [FGD with elderly men in Sabougou, Kolokani]

Social impact of LE on patients

LE can led to social isolation, and the use of hygiene measures can help reduce the stigma associated with this condition. Thus, a man in Kolondieba, during a focus group discussion, said: "*Some people feel disgusted with sick people like us and, except for a few, no one really cares about us"*. [FGD with elderly men in Kolondieba].

Another woman, during an individual interview, stated that:

"*Yes, even now people in my family think that I refuse to be a housekeeper, to prepare for the family, whereas it is very difficult for me to stand up for long periods of time*". [IDI with a woman from Kolondieba]

The use of hygiene measures has enabled some people to participate again to their communities' collective works. This is illustrated by a quote from a woman interviewed during a focus group discussion:

"*We used to have difficulty walking, but thanks to the LEDoxy study, I can walk long distances, and I can take part in collective activities*" [FGD with elderly women from Kolondieba].

Discussion

LE is one of the complications of LF, which is an NTD affecting many people in rural, remote, and hard-to-reach areas of Mali. The current study participants' median age was 56 years [25-70]. This age group corresponds to the professionally active age group (1). This age group is more frequently affected by LE because this chronic complication occurs many years after infection, and once it has occurred, it is almost irreversible without early and permanent local hygiene (2) or a reversal of the primary cause that could sometimes be resolved using lymphatic surgery. The high number of cases in this age group may be due to the beneficial effects of mass treatment with ivermectin and albendazole that protected younger age groups by reducing their exposure to LF parasites and therefore their likelihood to develop LE. The MDA was instituted in Kolondieba from 2005 and in Kolokani from 2006 (16). This result

may help draw the attention of political and financial decision-makers to the need to target the morbidity management and disability prevention (MMDP) efforts at these at-risk age groups. Most of the participants were farmers, 78.1%. This means that LE in Mali should be considered in rural areas as a serious threat to patients' productivity. In these areas, agriculture is the basis of subsistence and therefore pivotal to the local economy. In the Malian context, the schooling rate is usually low in rural areas. This implies that special attention must be paid to schooling for people living in rural areas to promote health education and improve the prevention and self-management of ADLA crises in these areas. These results provided valuable information on the socio-demographic characteristics of people affected by LE, which may help to develop tailored treatments and therapeutic approaches especially when it comes to sensitization and training materials developments since those should consider features such as the very low literacy rate.

Cost of LE management

As part of the LEDoxy study, LE carriers were trained in local hygiene use and provided with free-of-charge treatment at community health centres and, for the intervention group. The average cost of managing an ADLA crisis decreased significantly 16 months after LEDoxy. This suggests that washing limbs daily for a prolonged period reduced the frequency and severity of acute seizure episodes, resulting in lower management costs. The average monthly income of people living in these rural areas of Mali is about US $ 105.82 equivalent to 64,485 F CFA (17). The use of a basic package of care is a low-cost alternative for the prevention of ADLA crises. The use of this package reduces the frequency of attacks and therefore decreases the cost of LE management while the loss of time of work also decreases. Given the significant benefits of affected limb hygiene, it is important for LF elimination programs to implement and find ways to sustain this hygiene measure within LE patients for a better and effective prevention/management of LE (3). To do so, communicating about the economic

burden of LE would be useful to convince donors to support LF elimination programs for patients and their relatives in these poor environments. The morbidity management component through the implementation of community-based morbidity management programs would also be necessary to support the use of the basic package of care and further reduce the burden of disease. In addition, the WHO recommends further improvements for health facilities, such as ensuring the availability of antibiotic creams and tablets in health facilities located in endemic areas. In addition, it is essential to ensure the presence of qualified staff for the effective management of lymphedema cases in general, and ADLA attacks in particular. These measures strengthen the capacity of health facilities to respond to the specific medical needs of people affected by LE.

Productivity of LE patients

Some patients reported a positive effect of hygiene measures on their productivity. The costs associated with managing the ADLA account for the bulk of the expenditure, and a large part of the economic burden is due to the indirect costs associated with the loss of economic productivity (18). LE and ADLA may require patients to work fewer days per year or fewer hours per day, and they may have lower earnings because they cannot engage in work which may increase the risk of acute attacks (4,5). In advanced stages, LE is very disabling. In the intermediate stages, it results in partial disability with considerable loss of productivity, and the early stage progresses to the advanced stage in the absence of adequate management (6). Rigorous skin hygiene is essential to avoid superinfection, as the affected limb's skin is more vulnerable to infection (19). The basic package of care is effective in reducing the frequency and intensity of ADLA and thus helping to increase patients' productivity. Current results imply that hygiene measures are essential to mitigate the economic impact of LE, by reducing work absences and productivity losses. Therefore, policymakers can be advised to promote the implementation of LE management programs based on hygiene measures to reduce the

incidence of ADLA episodes and improve the quality of life (QoL) of LE patients. Additionally, stakeholders can also provide increased support for operational research to assess the effectiveness and feasibility of other LE management interventions, such as self-care protocols and strategies for integrating hygiene measures into the minimum package of activities of community health centres to improve LE management in remote LE-affected communities.

Social impact of lymphedema

In a focus group discussion, patients reported a feeling of social isolation related to LE. After the introduction of the basic package of care, more than half of the patients (76.5%) reported that they did not experience any social difficulties related to the condition. The physical, social, and psychological suffering caused by LE is considerable (7). The reduction of social difficulties using local hygiene measures is evidence of the effectiveness of this intervention (8,9).

LE patients in rural areas of Mali have few income-generating activities and most of them are older persons. Social difficulties such as stigma and reduced ability to perform daily activities lead to social isolation, which in turn contributes to depression and other health issues (10). In addition, an increase in the incidence of ADLA can hurt socioeconomic status due to work disability (11). This result highlights a significant decrease in social difficulties related to LE with regular use of local hygiene measures. However, further research is needed including studies testing large-scale interventions to develop a better understanding of the impact of the WHO-recommended basic package of care including local hygiene measures, physical exercise, antibiotic and antifungal topical use, and wound avoidance, to see how they will lead to improved quality of life and under what circumstances this care may be easiest (12). This requires an assessment of QoL based on a broader perspective including environmental determinants as well as the health centre frequented by patients in the context of continuing

education of rural health professionals and LE patients. Large-scale initiatives to reduce the social difficulties associated with LE must also be undertaken. This requires a clearer understanding of the process of occurrence of these difficulties faced by patients so that more effective interventions can be implemented. Social support by the community, local healthcare workers' assistance, and the donation of hygiene kits to patients with LE could significantly and sustainably decrease the social difficulties associated with this condition.

LE hygiene practice sustainability

some participants indicated that they no longer washed their affected limbs. In the absence of regular limb hygiene, patients resort to remedies and treatments that can be ineffective and costly. In the Malian context, traditional practices for the management of LE include herbal preparations applied to the affected limb, skin incision, and analgesics purchased from itinerant drug vendors outside of pharmacies. The basic package of care is an effective and less costly means for LE management (13). If all affected patients practice hygiene according to WHO recommendations, there would be significant benefits in terms of health, QoL, and economic productivity (14). To achieve this, a method based on the integration of LE management into the current health system and the organization of patients into self-support associations for improved follow-up care must be undertaken (20). Doing so would require local readaptation and tailoring that would consider local features and culture.

In rural Mali, the main activity is agriculture, which is highly impacted by the LE and related ADLA, so an effective and efficient program to manage LE must include the establishment of income-generating activities (IGAs), the types and modalities of which must be discussed and thought out by associations of people living with LE and by the community to make this action sustainable.

Limitations

Despite being original, this study faced some limitations including the design. It would be better to use the family mean income instead of the Guaranteed Interprofessional Minimum Wage which is much more relevant for those getting monthly salary because most of our participants are from rural areas. Moreover, recall bias can occur because of self-reported data in which participants might not remember or accurately report their socio-economic conditions before and after LEDoxy.

Conclusion

This study suggests that the use of a basic package of care significantly improved the socioeconomic conditions of LE carriers mainly by reducing the cost management of ADLA attacks. There is a need to establish a community-based lymphedema management program with income-generating activities as a strategy to support the sustainability of these measures.

Authors' contributions

All authors contributed to the design and development of the manuscript. HD developed the design framework for this proposal. AFD, LD, and SSD led the implementation of the survey. HD, DT, YIC, and SOT developed the initial idea for the article. DT, MS, ID, OC, SYC, and HD wrote the first draft of the manuscript. HS, SD, YIC, and TBN made critical contributions to the manuscript. All authors have read and approved the final version of the manuscript.

References

1. Mamadou G, Yamoussa K, Mariam D, Mamadou K, Koureissi T, Mélégué SA, et al. Aspects épidémiologiques et cliniques du lymphøedème dans le service de dermatologie du CHU Gabriel TOURE, Bamako. Health Sci Dis. 2019;20(1):73-6.

2. Pfarr KM, Debrah AY, Specht S, Hoerauf A. Filariose et lymphøedème. Immunol Parasites. nov 2009;31(11):664-72.

3. Weiss R. Cost of a lymphedema treatment mandate - 16 years of experience in the Commonwealth of Virginia. Health Econ Rev. 23 juill 2022;12(1):40.

4. Eneanya OA, Garske T, Donnelly CA. The social, physical and economic impact of lymphedema and hydrocele: a matched cross-sectional study in rural Nigeria. BMC Infect Dis. 23 avr 2019;19(1):332.

5. Roberson ML, Strassle PD, Fasehun LKO, Erim DO, Deune EG, Ogunleye AA. Financial Burden of Lymphedema Hospitalizations in the United States. JAMA Oncol. 1 avr 2021;7(4):630-2.

6. Sawers L, Stillwaggon E. Economic Costs and Benefits of Community-Based Lymphedema-Management Programs for Lymphatic Filariasis in India. Am J Trop Med Hyg. juill 2020;103(1):295-302.

7. Litt E, Baker MC, Molyneux D. Neglected tropical diseases and mental health: a perspective on comorbidity. Trends Parasitol. mai 2012;28(5):195-201.

8. Omidi Z, Kheirkhah M, Abolghasemi J, Haghighat S. Effect of lymphedema self-management group-based education compared with social network-based education on quality of life and fear of cancer recurrence in women with breast cancer: a randomized controlled clinical trial. Qual Life Res Int J Qual Life Asp Treat Care Rehabil. juill 2020;29(7):1789-800.

9. Turner HC. Health economic analyses of the Global Programme to Eliminate Lymphatic Filariasis. Int Health. 22 déc 2020;13(Suppl 1):S71-4.

10. Stillwaggon E, Sawers L, Rout J, Addiss D, Fox L. Economic Costs and Benefits of a Community-Based Lymphedema Management Program for Lymphatic Filariasis in Odisha State, India. Am J Trop Med Hyg. 5 oct 2016;95(4):877-84.

11. Ridner SH. The psycho-social impact of lymphedema. Lymphat Res Biol. 2009;7(2):109-12.

12. Dolo H, Coulibaly YI, Konipo FN, Coulibaly SY, Doumbia SS, Sangare MB, et al. Lymphedema in three previously Wuchereria bancrofti-endemic health districts in Mali after cessation of mass drug administration. BMC Infect Dis. 15 janv 2020;20(1):48.

13. Horton J, Klarmann-Schulz U, Stephens M, Budge PJ, Coulibaly Y, Debrah A, et al. The design and development of a multicentric protocol to investigate the impact of adjunctive doxycycline on the management of peripheral lymphoedema caused by lymphatic filariasis and podoconiosis. Parasit Vectors. 30 mars 2020;13(1):155.

14. Dolo H, Coulibaly YI, Konipo FN, Coulibaly SY, Doumbia SS, Sangare MB, et al. Lymphedema in three previously Wuchereria bancrofti-endemic health districts in Mali after cessation of mass drug administration. BMC Infect Dis. janv 2020;20(1):48.

15. Horton J, Klarmann-Schulz U, Stephens M, Budge PJ, Coulibaly Y, Debrah A, et al. The design and development of a multicentric protocol to investigate the impact of adjunctive doxycycline on the management of peripheral lymphoedema caused by lymphatic filariasis and podoconiosis. Parasit Vectors. 30 mars 2020;13(1):155.

16. World Health Organization = Organisation mondiale de la Santé. Global programme to eliminate lymphatic filariasis: progress report, 2017 – Programme mondial pour l’élimination de la filariose lymphatique: rapport de situation, 2017. Wkly Epidemiol Rec

Relevé Épidémiologique Hebd [Internet]. 2 nov 2018 [cité 12 août 2022];93(44):14-602. Disponible sur: https://apps.who.int/iris/handle/10665/275720

17. INSTAT. Institut National de la Statistique du Mali|INSTAT. [cité 3 sept 2023]. Enquête Régionale Intégrée sur l'Emploi et le Secteur Informel (ERI-ESI) au Mali. Disponible sur: https://www.instat-mali.org/fr/publications/enquete-regionale-integree-sur-lemploi-et-le-secteur-informel-eri-esi-au-mali

18. Sawers L, Stillwaggon E. Economic Costs and Benefits of Community-Based Lymphedema-Management Programs for Lymphatic Filariasis in India. Am J Trop Med Hyg [Internet]. 8 juill 2020 [cité 2 oct 2023];103(1):295-302. Disponible sur: https://ajtmh.org/doi/10.4269/ajtmh.19-0898

19. Bernas M, Thiadens SRJ, Smoot B, Armer JM, Stewart P, Granzow J. Lymphedema following cancer therapy: overview and options. Clin Exp Metastasis [Internet]. août 2018 [cité 2 oct 2023];35(5-6):547-51. Disponible sur: http://link.springer.com/10.1007/s10585-018-9899-5

20. Semrau M, Ali O, Deribe K, Mengiste A, Tesfaye A, Kinfe M, et al. EnDPoINT: protocol for an implementation research study to integrate a holistic package of physical health, mental health and psychosocial care for podoconiosis, lymphatic filariasis and leprosy into routine health services in Ethiopia. BMJ Open. 15 oct 2020;10(10):e037675.

List of abbreviations

ADLA: Acute adenolymphangitis attack

GPELF: Global Program for the Elimination of Lymphatic Filariasis

LE: Lymphedema

LF: Lymphatic Filariasis

MDA: Mass drug administration

NLFEP: National Lymphatic Filariasis Elimination Program

WHO: World Health Organization

Statements

All manuscripts must contain the following sections under the heading "Statements

Consent for publication

Not applicable.

Competing interests

The authors declare that they have no conflict of interests. Author Details

Availability of data and materials

The data used in this manuscript can be found through the International Center of Excellence in Research of the University of Science, Techniques and Technologies of Bamako by sending an email to the principal investigator and first author of the manuscript.

Funding

This study was funded by the University of Sciences, Techniques and Technologies of Bamako through the research promotion grant to the teaching and research professors awarded to HD. In addition, the study was funded in part (to TBN) by the Division of Intramural Research (DIR) of the National Institute of Allergy and Infectious Diseases, NIH.

Acknowledgments

We would like to thank the National Lymphatic Filariasis Elimination Program for its collaboration in implementing this study. We thank the health districts officers of Kolondieba and Kolokani for making it possible to carry out this study as well as the community health

workers. Our acknowledgments go to the US Agency for International Development, through the Task Force for Global Health, Decatur, Georgia, USA, for funding the main study (Ledoxy). We thank Ledoxy study team for sharing the participants data to support the embedded current study. Special thanks to the University of Sciences, Techniques and Technologies of Bamako for funding this study.

Author's contact information (optional)

Housseini Dolo: hdolo@icermali.org ; Diadje Tanapo: diadje.tanapo@icermali.org ; Sekou Oumarou Thera: sekouthera@icermali.org; Moussa Sangaré: mbsangare@icermali.org; Abdoul Fatao Diabaté: afatao@icermali.org; Lamine Diarra: lamdiarra@icermali.org; Salif Sériba Doumbia: salifdoumbia@icermali.org; Siaka Yamoussa Coulibaly: yamoussa@icermali.org; Ilo Dicko: ilodicko@icermali.org; Oumar Coulibaly : oumar.coulibaly@icermali.org; Ousmane Faye: fayeo@yahoo.fr; Hamadoun Sangho: prsangho@gmail.com; Seydou Doumbia : sdoumbi@icermali.org; Yaya Ibrahim Coulibaly: yicoulibaly@icermali.org; Thomas B. Nutman: tnutman@niaid.nih.gov

Figure 1

Click here to access/download;Figure;Fig 1.tif

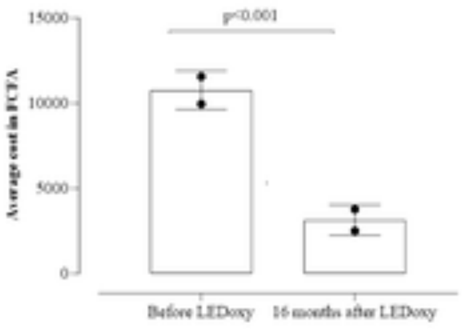

Checklist

Click here to access/download
Supporting Information
PLOSOne_Human_Subjects_Research_Checklist .pdf

IRB approval_original

Click here to access/download
Supporting Information
IRB_approval _let_original.pdf

IRB approval_translate_english

Click here to access/download

Supporting Information

IRB_approval_let translate_English.pdf
