# Supplementary material for: Implementation of basic package of care improved socio-economic conditions of lymphedema patients in rural Mali after two years follow-up
Source: PLOS Glob Public Health. 2025 Nov 11;5(11):e0005454. doi: 10.1371/journal.pgph.0005454 (PMC12604757; doi:10.1371/journal.pgph.0005454)
Supplement: Attachment — Submitted filename: Point by point response to Review Lymphoedema paper .docx [file pgph.0005454.s005.docx]

**Point by point responses to reviewers’ comments**

**Bamako, 23 June 2025**

On the behalf of the co-author, I as corresponding author, sincerely thank the reviewers for their detailed and productive feedback. We believe the suggested revisions have significantly strengthened the manuscript, enhancing its clarity, methodological rigor, and overall scientific merit. Our point-by-point responses are detailed below.

**Comment on editor email**

Congratulations to the authors for this very important manuscript which clearly depicts the importance of essential package of care in reducing the acute ADL attacks and thereby improving the quality of life of patients with chronic lymphedema and the improvement in socioeconomic situation and study itself will help this to be an important advocacy point for continuing MMDP and arriving at GPELF goals

We thank for mentioning that this very important manuscript

Even though Ledoxy study was mentioned, there seems to be agap to understand the relation between the two studies. So a short summary of the study will help readers to understand this well.

A short summary of Ledoxy study has been added to introduction for understanding

It would be good to mention about the Universal Health coverage in the context of patient care and self-care.

The introduction section has been edited to introduce the universal health coverage (UHC) (page 5, line 140 to 146)

**Overall Impression**

This manuscript " Implementation of Basic Package of Care Improved Socio-Economic Conditions of Lymphedema Patients in rural Mali after two years follow-up" addresses a highly pertinent public health issue; the socio-economic impact of lymphedema (LE) and the effectiveness of the Basic Package of Care (BPC) in rural, endemic settings. The study's findings, highlighting a substantial reduction in acute filarial attacks (ADLA) frequency and management costs, coupled with positive reports on reduced social isolation, stigma, and improved productivity, are compelling and advocate strongly for the sustained implementation of these care programs. The mixed-methods approach is a notable strength, offering both quantitative evidence and invaluable qualitative insights into patients' lived experiences.

However, the manuscript, in its current form, requires significant revisions to enhance methodological clarity, analytical rigor, and adherence to scholarly conventions. The abstract's description of the study design is imprecise and needs to be clarified. Details regarding data collection timelines and the handling of outliers should be explicitly stated for transparency.

Furthermore, the discussion section, while insightful, needs more critical engagement with the literature. Claims, particularly concerning age group prevalence, irreversibility of LE, and the role of traditional practices, require more robust and accurately cited evidence. Consistency in citation style and precise statistical notation are also essential. The organization of the discussion, with its fragmented subheadings, could be improved by transitioning to a more fluid, paragraph-based thematic flow. Addressing these points will significantly strengthen the manuscript's scientific merit and impact.

We appreciate the reviewer's full assessment of our manuscript. We are happy that the reviewer recognizes the public health pertinence, compelling findings, and the strength of our mixed-methods approach. We have carefully addressed all the points raised to enhance clarity, rigor, and adherence to academic conventions. The specific revisions made in response to these overarching comments are detailed in the << Specific Comments>> section below.

**Specific Comments**

**Abstract**

Line 27 to Line 28: A “local hygiene package”? and “quality of life”? Can you be more specific? Especially for the quality of life, which aspects?

Thank you for your feedback. We have edited the abstract to provide the requested clarification:

Revised version (Abstract, lines 28-31): “A local hygiene package (washing limbs, wound care, physical exercise) has previously been demonstrated to be effective at improving lymphedema (LE) patient’s quality of life particularly by reducing adenolymphangitis attacks, medical costs, social isolation, and stigma, while restoring their ability to work.”

Line 30 to Line 32: A “cross-sectional study” was used, but the authors mentioned “before and after approach” which is pointing towards a longitudinal design with two data collection points rather than cross-sectional that measures one data point. The title suggests this is a follow-up study (may also be a cohort design), or was this an interventional study (quasi experimental design)? Please make it clearer. Also, was the data purely primary, secondary, or a combination of both?

We thank the reviewer for this important clarification regarding the study design. We have revised the abstract to accurately reflect the cross-sectional nature of the study, which collected retrospective data on pre-intervention conditions and prospective data on post-intervention conditions at a single point in time. We have also clarified that all data were primary. (See page 2 line 39 to 44)

Line 32 to Line 35: Mention the time period when the study was done.

Revised version, Line 39 to Line 40: “We conducted this study in the health districts of Kolondieba and Kolokani from September to December 2021”

Line 36: 196 LE patients were enrolled “median 56 years [27-70] years” is what? An interquartile range or range?

The notation “median age of 56 [25-70]” indicates the range (minimum-maximum) of ages, not the interquartile range

Revised version, Line 47: “We investigated 196 LE patients with a median age of 56 years [range: 25-70 years]”.

Line 38: and Line 40: (p<10-3)? Is this a P-Value? If so, edit accordingly (example p < 0.05 is a more standard way of writing it)

We changed (p < 10-3) to (p < 0.05). (see page 3, line 89 )

Revised version, Line 47 to Line 49: “We observed a reduction in the monthly frequency of acute filarial attacks (ADLA) from 90.8% (178/196) before the trial to 43.9%, (86/196) after the trial (p<0.05)”

Line 44 to Line 45: This sentence is not clear. Please make clearer.

Revised version, Line 84 to Line 85: “Therefore, a community-based lymphedema management program supported income-generating activities is needed for long-term sustainability”. ( see page 3, line 86 to 87)

**Main Article**

**Introduction**

Ln 49 and Ln 50: Lymphedema (LE) and Acute adenolymphangititis (ADLA) introduction is good following a funnel approach.

We have placed the reference at the end of the sentence to maintain consistency.

Revised version, Line 96 to Line 97: “Lymphedema (LE) is a chronic swelling of a limb due to an accumulation of lymphatic fluid caused by an obstruction or dysfunction of the lymphatic system (1). In the context of neglected tropical diseases, it is mainly associated with lymphatic filariasis (LF) (2). Acute adenolymphangitis attacks (ADLA) is an acute inflammatory episode occurring in pre-existing lymphedema, characterized by bacterial infection of the lymphatic vessels. It manifests as fever, intense pain, skin redness, and sudden worsening of oedema (3)”. (see page 4)

Ln 68 – Ln 70. Maintain consistency with citation at the end of the sentence as done previously.

We have placed the reference at the end of the sentence, as was done previously.

Revised version, Ln 137 – Ln 139 : “In Mali, based on a previous study on LE in three health districts, the majority of people with LE were living in remote rural areas (15)”.

Ln 74 – Ln 76: “During the LEDoxy study…” in-text citation only included WHO (14), (13) was not included.

We have added reference (16).

Revised version, Ln 149 – Ln 151: “During LEDoxy, all subjects received hygiene kits and training on self-management of LE as recommended by WHO (16,17)”.

Line 76 to Line 81: Was this still talking about the LEDoxy study? Please add the citation and make this clearer.

Revised version Line 142 to Line 148: We have added reference (16), LEDoxy reference (see page 5)

Line 81: The font looks smaller/different, adjust to match the rest if the article.

We have adjusted the font size for the text. (see page 5)

**Methods**

Line 83 to Line 89: Started with ethical consideration. Consider following the journal guidelines for the arrangement.

We have modified the "Ethical Considerations" section

Revised version, Line 160 to Line 190: This study was approved by the Ethics Committee of the University of Science, Techniques and Technology of Bamako (USTTB), Mali (Approval Number: 2021/231/USTTB). Written informed consent was obtained from all participants prior to enrolment. For participants unable to write, fingerprint consent was obtained in front of a witness in accordance with procedures approved by the Ethics Committee. Participants received full verbal explanations of the study objectives, procedures, risks/benefits, and their right to withdraw without penalty, in the local language (Bambara). All data were anonymized, stored on encrypted servers with password protection, and accessible only to authorized investigators. As compensation, all participants received their transportation costs (a lump sum of 3,000 FCFA around US $ 5.25). (see page 5-6)

Line 91: Please refer to the previous comment on line 30 to line 32 of the abstract.

We have modified it in accordance with the previous comment.

Revised version, Line 192 to Line 199: “We conducted a cross-sectional study embedded in “LEdoxy” study participants. The same questionnaire was used in the same trial participants to collect retrospective data on their pre-trial conditions and prospective data on post-trial conditions at the same time. Primary data were collected using a digital questionnaire and qualitative methods, including in-depth interviews and focus group discussions guided by a pre-established interview guide, without recycling 'LEDoxy' data. We conducted this study in the health districts of Kolondieba and Kolokani from 26 September to 25 December 2021, using mixed methods (quantitative and qualitative) data collection. ”. (see page 6)

Line 96 to Line 107: For the study population consider mentioning the age range and sex of the study population. The sampling frame is also not clear; is the sampling frame the health districts or the LE patients? Is so, did the authors have any exclusion criteria? Write about the sampling for the quantitative and qualitative data collection on separate paragraphs for clarity. Elaborate on the sample size calculation and the sampling technique and procedure. Description of the sampling technique and procedure is not clear.

Thank you for your constructive comments regarding the "Study Population" section. We have completely revised this section to provide the requested clarifications. The following changes have been incorporated:

“The study population consisted of patients of both sexes with lymphedema who had previously been recruited for the LEDoxy clinical trial (NCT02927496) conducted from June 27, 2018, to December 19, 2020. The Kolondieba and Kolokani health districts were selected because of: (i) their documented endemicity of lymphatic filariasis, (ii) their high density of recorded lymphoedema cases, and (iii) the patient follow-up mechanisms put in place as part of the LEDoxy trial (15,18).

The inclusion criteria were as follows: confirmed participation in LEDoxy, residence in the study area for ≥ 6 months prior to data collection, age ≥ 18 years, and voluntary informed consent. The exclusion criteria were as follows: individuals who did not give their consent and participants who had moved outside the study districts.

Quantitative component sampling

We used exhaustive sampling by inviting the 220 former LEDoxy participants from both districts. This approach ensured maximum statistical power and representativeness of the initial trial cohort. All eligible patients were contacted and 196 participated (response rate: 89.1%).

Qualitative component sampling

Convenience sampling was used to recruit participants. We determined the sample size (8 FGDs; 10 IDIs) based on the principles of data saturation and operational feasibility. Per district, we conducted: 4 FGDs stratified by gender (men/women) and age group (youth/adults); 5 IDIs with targeted volunteers representing different stages of LE and different socioeconomic backgrounds. A total of eight FGDs and ten IDIs were successfully conducted.” ( line 201 to 243 , see page 6-7)

Please, see Line 194 to Line 227 for the revised version.

**We have mentioned the age group and sex ( see line 227)**

**Clarification of the sampling frame**

District selection: Based on: (i) Documented endemicity of lymphatic filariasis, (ii) High density of lymphedema cases, (iii) Existing follow-up of the LEDoxy trial,

Patient selection: From the LEDoxy trial (NCT02927496)

**Exclusion criteria added:** Non-consenting individuals**,** Participants who left the study districts

**Quantitative/qualitative separation:** Quantitative component sampling, Qualitative component sampling (see line 230 to 240)

Line 107: ‘A total of eight FGDs and ten IDIs were planned’ This has been done already please edit.

Thank you for highlighting this point. The sentence in question has indeed been modified in the revised version to reflect the completion of interviews rather than simply their scheduling.

Revised version, Line 235 to Line 240: "A total of eight FGDs and ten IDIs were successfully conducted.

Questionnaire design and administration

Line 109 to Line 110: “conceived”. Rephrase sentence that may be confusing. I understand that an electronic version of the survey questionnaire was used for data collection. And based on line 112 to line 113, the questionnaire was standardized through the process of pre-testing, correction, and validation. Also interviewers were trained. It is safe to edit line 109 to line 110 to capture the fact that an electronic version of the standardized survey questionnaire was used to collect data. (Add clarity to this)

Thank you for highlighting this potential point of confusion. The sentence has been revised for clarity as follows:

Revised version, Line 242 to Line 243: “A digital version of the standardized survey questionnaire was developed and implemented using the Kobo Toolbox platform for electronic data collection via smartphones”. (see page 7)

Line 114: mentions kobotoolbox was used. Mentioning this once is sufficient.

Thank you for this helpful suggestion. We have made the requested change by removing the redundant reference to Kobotoolbox.

Revised version, Line 247 to Line 248: “Field collected data were checked and edited in real-time by the supervisors and the data manager.”

Line 121: “Economic Evaluation” was not captured in the abstract or the objectives. Please check to ensure it is captured in all sections.

Thank you for highlighting this important point of methodological consistency. We did indeed fail to explicitly mention the economic evaluation in certain key sections. The following changes have been made to ensure per section consistency:

Abstract (Addition): Revised version, Line 35 to Line 38: “This study aimed to assess the socio-economic impact including an **economic evaluation** of the costs of car, of regular local care based on the hygiene package used among LE patients”. (see page 2)

Introduction (Addition): Revised version, Line 152 to Line 157: “Thirty-six months after the study completion, the current study aimed to assess the socio-economic impact with an **economic evaluation** of the costs of managing ADLA of implementing a basic package of care in the management of LE in the two rural study health settings to fill the knowledge gap on the socio-economic impact of the WHO-recommended basic package of care in remote rural communities.”

Data analysis

Ln 134: “p value =<0.05…” use the appropriate symbol (example ≤).

Thank you for pointing out this statistical scoring inaccuracy. The correction has been applied as follows: Ln 291: “Any p values ≤0.05 were considered statistically significant” (see page 8).

**Results**

Table 1: remove the bold horizontal borderlines separating the variables of the sociodemographic characteristics. Leave only the borderline for the headings and after the last row.

We removed the bold horizontal borders separating the variables from the sociodemographic characteristics and kept only the border for the titles and after the last line. (see page 9)

Table 2: see comment for table 1.

We did the same for table 2. (see page 10- 11)

Figure 1 is tiny and not visible. Adjust.

We adjusted Figure 1

Generally for the results, I suggest you should create major subheadings for the presentation of quantitative results and for the qualitative results to guide the reader.

We have created main subheadings for the presentation of quantitative results and for qualitative results (revised version line 296 page 8) and ( revised version line 331 page 11)

**Discussion**

Line 220 to Line 221: median age… also indicate if [25-70] is range or interquartile range?

Thank you for this important clarification regarding the presentation of demographic data. The [25-70] notation has indeed been clarified as follows:

Revised version, Line 386 to Line 389: “The current study participants’ median age was 56 years [range: 25-70 years]”.

Line 221: “This age group corresponds to the professionally active age group (1)”. The statement may not be valid. I checked the reference; it is an article in French as such could not verify. Please check. What is the age of retirement in Mali? I was able to see age 60 years and below, please confirm.

Thank you for this insightful observation regarding the characterization of the professionally active age group in Mali. We have modified the wording to more accurately reflect Mali's socioeconomic reality:

Revised version, Line 386 to Line 389: “In the rural context of Mali, where subsistence agriculture predominates, people in this age group (25-70 years old) generally remain economically active, regardless of official retirement thresholds, as agricultural work continues based on physical capacity rather than chronological age”

Clarification on retirement:

- Although the legal retirement age is indeed 60 for the formal sector in Mali ([Code de Sécurité Sociale, 2012](https://www.ilo.org/dyn/natlex/docs/ELECTRONIC/94525/110351/F-1118513553/MLI-94525.pdf)),
- In rural areas (98% of our sample), 80% of those over 60 continue to work in agriculture ([Enquête EHCVM, 2019](https://www.instat-mali.org/publications/ehcvm)). (see page 13)

Line 222 to Line 225: maintain the consistency of the in-text citations at the end like it was done in most of the article. The reference no. 2. In the reference list refers to the abstract of the study not the full text. The full text (Pfarr KM, Debrah AY, Specht S, Hoerauf A. Filariasis and lymphoedema. Parasite Immunol. 2009 Nov;31(11):664-72. doi: 10.1111/j.1365-3024.2009.01133.x. PMID: 19825106; PMCID: PMC2784903.) does not support the claims in the authors’ sentence “This age group is more frequently affected by LE because this chronic complication occurs many years after infection, and once it has occurred, it is almost irreversible without early and permanent local hygiene (2) or a reversal of the primary cause that could sometimes be resolved using lymphatic surgery.” Please check and edit your citation.

We have maintained consistency of in-text citations at the end of the sentence. (see this changes across section of the article)

**Line 102:** We have changed the reference (Pfarr KM, Debrah AY, Specht S, Hoerauf A. Filariasis and lymphoedema. Parasite Immunol. 2009 Nov;31(11):664-72. doi: 10.1111/j.1365-3024.2009.01133.x. PMID: 19825106; PMCID: PMC2784903.) to (Rockson SG. Advances in Lymphedema. Circ Res. 2021 Jun 11;128(12):2003-2016. doi: 10.1161/CIRCRESAHA.121.318307. Epub 2021 Jun 10. PMID: 34110905. ) (see reference section page 20 line 593).

Line 225 to Line 227: “The high number of cases in this age group may be due to the beneficial effects of mass treatment with ivermectin and albendazole that protected younger age groups by reducing their exposure to LF parasites and therefore their likelihood to develop LE.” Please provide citation.

**Line 395:** We added an additional reference to support the hypothesis regarding the impact of mass treatments on the age distribution of lymphedema cases. “Ichimori K, King JD, Engels D, Yajima A, Mikhailov A, Lammie P, Ottesen EA. Global programme to eliminate lymphatic filariasis: the processes underlying programme success. PLoS Negl Trop Dis. 2014 Dec 11;8(12):e3328. doi: 10.1371/journal.pntd.0003328. PMID: 25502758; PMCID: PMC4263400.”

Line 233 to Line 234: “In the Malian context, the schooling rate is usually low in rural areas.” Is this referring to findings from the study? Please make it clearer.

Revised version, **Line 404 to Line 407:** “In our sample, 90.8% (178/196) of participants had received no formal education (Table 1), reflecting the national reality where the net enrollment rate in rural areas was 68.3% for primary education in 2020, according to the National Institute of Statistics of Mali”. (see page 14)

Line 270: “In advanced stages, LE is very disabling” Provide citation for this.

Line 467: We have added an appropriate quote to support the claim regarding disability in advanced stages of lymphedema “WHO. (2011). Monitoring and epidemiological assessment of mass drug administration in the global programme to eliminate lymphatic filariasis. Geneva, pp. 12-14, Disponible en ligne : https://apps.who.int/iris/handle/10665/44772) »

Line 311: starts with small letter “s” please edit.

Line 518: We have corrected this typographical error. (see page 17).

Line 311 to Line 315: “In the absence of regular limb hygiene, patients resort to remedies and treatments that can be ineffective and costly. In the Malian context, traditional practices for the management of LE include herbal preparations applied to the affected limb, skin incision, and analgesics purchased from itinerant drug vendors outside of pharmacies.” Provide citation if these are not findings from your study.

Thank you for your request for clarification. The practices described in this paragraph are taken directly from our qualitative interviews and therefore constitute original findings from this study. The wording has been changed as follows to make this explicit:

Revised version, Line 518 to Line 522 : “According to qualitative data collected during individual and group interviews, in the absence of regular limb hygiene, patients resort to traditional remedies that are sometimes ineffective and costly. The practices reported included: applying herbal preparations to the affected limb, making incisions in the skin to ‘drain the edema,’ and purchasing painkillers from street vendors." (see page 18).

Limitations

Elaborate on the limitations in design; your sample size, sampling (if probability sampling was not used), also mention if your results are not generalizable at a National or district level as a limitation. Also mention the strengths of your study before the limitation.

Thank you for these suggestions, which reinforce the rigor of our section on limits. We have restructured this section as follows:

Revised version, Line 536 to Line 539: “This mixed-methods study provides data exclusively from rural areas where lymphatic filariasis was endemic, based on primary data collection. Quantitative rigor (n = 196, or 89.1% of the initial cohort) was complemented by rich qualitative information (8 group discussions, 10 individual interviews).

However, the study has some limitations. First, non-probability sampling (exhaustive for quantitative components, convenience sampling for qualitative components) may limit generalizability. Second, the results apply specifically to similar rural contexts where LF is endemic, but not to urban contexts. Third, the use of the guaranteed minimum wage as an economic reference was not optimal for agricultural populations (79.1%). Finally, memory bias may affect self-reported socioeconomic data before and after the intervention”.

I suggest the subheadings (Cost of LE management, productivity of LE patients, social impact of lymphedema, LE hygiene practice sustainability, and Limitations) in the discussion should be eliminated. Start discussion a new idea or theme on a new paragraph, this will improve the flow.

We have removed the subtitles from the discussion. (see revised discussion section)

Generally, check your references ensure they are all in English as you submitted your manuscript in English.

All references are in English. (see the revised references section)
