# Supplementary material for: Implementation of basic package of care improved socio-economic conditions of lymphedema patients in rural Mali after two years follow-up
Source: PLOS Glob Public Health. 2025 Nov 11;5(11):e0005454. doi: 10.1371/journal.pgph.0005454 (PMC12604757; doi:10.1371/journal.pgph.0005454)
Supplement: Attachment — Submitted filename: Responses to reviewers- editor.docx [file pgph.0005454.s006.docx]

PGPH-D-25-00707R2
Implementation of Basic Package of Care Improved Socio-Economic Conditions of Lymphedema Patients in rural Mali after two years follow-up.
Dr Housseini Dolo

Dear Dr. Dolo,

Thank you for submitting your manuscript entitled "Implementation of Basic Package of Care Improved Socio-Economic Conditions of Lymphedema Patients in rural Mali after two years follow-up." to PLOS Global Public Health. Your manuscript files have been checked in-house, but before we can proceed we need you to address the following issues:

Just to point that this manuscript has long history with PlOS Global Public Health. Many editor where changed in the mean time. Please as new editor in charge with cheick all the submission / decision and revisons.

1. We note your Data Availability statement as follows:

“All relevant data are within the paper. However, de-identified data of this study can be requested per the University of Sciences, Techniques and Technologies of Bamako data sharing regulation.”
I replaced the orignal text with this quote
Please also provide non-author contact information (phone/email/hyperlink) for a data access committee, ethics committee, or other institutional body to which data requests may be sent. If applicable, please also provide any necessary information which interested researchers would need when requesting access to data in order to obtain the minimal data set for your study.

I added the email and tel contact of the permanent secretary of USTTB IRB as request sending contact.
